# Investigation of the Effects of Some Cardiovascular Drugs on Angiogenesis by Transgenic Zebrafish

**DOI:** 10.1155/2023/1958046

**Published:** 2023-04-24

**Authors:** Hui Lv, Bo Liu, Yongwen Qin

**Affiliations:** ^1^Department of Cardiovascular Disease, The Second Affiliated Hospital of Shanxi Medical University, Taiyuan, Shanxi 030001, China; ^2^Department of Cardiovascular Disease, Xinhua Hospital Affiliated to Shanghai Jiaotong University School of Medicine, Shanghai 200433, China; ^3^Department of Cardiovascular Disease, Changhai Hospital Affiliated to The Second Military Medical University, Shanghai 200433, China

## Abstract

**Introduction:**

Angiogenesis contributes to the pathophysiology of cardiovascular disease (CVD). Some cardiovascular drugs used in the treatment of CVD have an effect on the process of angiogenesis.

**Methods:**

Transgenic Tg (flk1: EGFP) zebrafish embryos were used to identify the effects of some cardiovascular drugs on angiogenesis during vertebral development *in vivo*. Zebrafish embryos at a one-cell stage or two-cell stage were cultured with embryo medium containing cardiovascular drugs at a final solvent concentration of 0.5% (V/V) dimethyl sulfoxide (DMSO) for 24 hours in 24-well plates.

**Results:**

We found that 6 drugs including isosorbide mononitrate, amlodipine, bisoprolol fumarate, carvedilol, irbesartan, and rosuvastatin calcium may affect angiogenesis by vascular endothelial growth factor (VEGF) signaling pathway.

**Conclusion:**

These new findings of some cardiovascular drugs should improve the treatment of cardiovascular diseases.

## 1. Introduction

Angiogenesis is an important part of the pathophysiology of cardiovascular diseases (CVD) [1–3]. Among various cardiovascular drugs commonly used to treat CVD, some are known to affect the process of angiogenesis. For instance, statins [4], which are widely used in the treatment of hyperlipidemia and coronary heart disease (CHD), can inhibit angiogenesis and reduce the rate of revascularization. Nifedipine [5], a calcium antagonist used to control hypertension, can induce human coronary artery endothelial cells to form capillary-like tubes and increase the total capillary density of the hamster-dilated cardiomyopathic heart. Besides, angiotensin-converting enzyme inhibitor (ACEI) can induce angiogenesis through upregulating fibroblast growth factor-2 (FGF-2) in coronary endothelium [6]. Moreover, SH-containing ACEI zofenoprilat triggers angiogenesis by improving the availability of hydrogen sulfide [7]. However, whether other cardiovascular drugs have this angiogenic or antiangiogenic activity is unclear.

In order to study angiogenesis *in vivo*, zebrafish (*Danio rerio*) provides an excellent model for the transparency of its embryo, which can direct observe vertebral development [8]. Transgenic zebrafish, such as zebrafish with green fluorescence protein (GFP) gene, shows strong GFP expression in vascular endothelial cells which can visually screen vascular pattern defects and observe the changes of vasculogenesis [9]. In this study, we used transgenic zebrafish as a model to test the angiogenesis or antiangiogenesis effects of 6 cardiovascular drugs with known pharmacological activities in the human body (Table 1).

## 2. Materials and Methods

### 2.1. Embryo Collection

Transgenic zebrafish were raised following standard care and maintenance protocols of a 14 : 10 light : dark cycle [10]. Embryos were obtained by natural spawning, staged according to established criteria [11], and raised in the embryo culture medium E3M (containing 5 mM NaCl, 0.17 mM KCl, 0.33 mM CaCl_2_, 0.33 mM Mg_2_SO_4_, 0.7 mM HEPES, and 10^−5^% methylene blue (pH 7.2) with a conductivity of 672 *μ*S and dissolved oxygen of 8.2 mg/l) in an incubator at 28.5°C. All the assays were performed at 25-28°C. Animal experiments were carried out in accordance with the guiding principles of the Animal Experimentation Ethics Committee of Second Military Medical University.

### 2.2. Drug Treatment

Seventeen cardiovascular drugs with known pharmacological activities in the human body were selected for the test (Table 1). The reagents were dissolved in DMSO and added to the embryo medium at a final DMSO concentration of 0.5%. One-cell stage or two-cell stage embryos were immersed in embryo medium containing drugs in 0.5% (*V*/*V*) DMSO final solvent concentration for 24 hours in a 24-well plate. Each compound was tested at six concentrations according to human blood concentration (Table 1). We used 20 embryos in each group to obtain higher throughput and less variability.

### 2.3. Angiogenic Function Assessment

In order to improve the screening process and better demonstrate the blood vessels in embryos, we used a stable transgenic Tg (fli-1: EGFP) zebrafish embryo, in which GFP was expressed in all endothelial cells of the vasculature in the intersegmental blood vessels (ISVs). Embryos were exposed to a single concentration of reagent for 24 hours and then dechorionated and fixed in methylcellulose (0.5%, m/V). After fixation, the vascular development of embryos was examined by a LEICA 205FA microscope. Antiangiogenic and angiogenic effects are defined as significant inhibition or enhancement of mature ISV formation, which normally connects the dorsal aorta and dorsal longitudinal anastomotic vessel (DLAV) in living embryos. The length of ISVs was calculated by LEICA 205FA software through point-to-point distance. The number of ISVs was calculated from photos taken under a microscope. ISV length and number were used to quantify angiogenesis.

### 2.4. Cell Line and Cell Culture

Human Umbilical Vein Endothelial Cells (HUVECs) (ATCC, Manassas, USA) were cultured in DMEM media (Invitrogen) with 2% fetal bovine serum (FBS), 100 U/ml penicillin and 100 *μ*g/ml streptomycin at 37°C with 5% CO_2_. Then, HUVECs were passaged 3 times a week.

### 2.5. Quantitative Reverse Transcriptase-Polymerase Chain Reaction (qRT-PCR)

The VEGF expression level in HUVECs was determined by qRT-PCR. Briefly, total RNA was extracted from 1 × 10^5^ HUVECs from passages 3 using TRIzol reagent (Takara Biotechnology, Dalian, China). Besides, NanoDrop 2000 (Thermo Fisher Scientific, Waltham, MA, USA) was utilized to analyze the quality and integrity of RNA. Next, 2 *μ*g RNA of each sample was reverse transcribed into cDNA by the PrimeScript™ 1st Strand cDNA Synthesis Kit (Takara Biotechnology). Subsequently, the amount of target RNA was normalized to that of internal control (GAPDH) and given by 2^−△△Ct^ relative to the control sample. Primers used in qRT-PCR were listed as follows (shown 5′-3′): VEGF F: TGGACCCTGGCTTTACTGCTG and R: GGCAATAGCTGCGCTGGTAGA; GAPDH F: GAEAACTTTGGCATCGTGGA and R: TGCAGGGATGATGTTCTGG.

### 2.6. Statistical Analysis

The data of the current study were analyzed using a mixed design analysis of variance between-subject factors of drugs, drug dose, and timing of drug administration. All experiments were repeated for at least three times. Next, the values were presented as mean ± standard definition (SD). And statistical analysis was performed with Microsoft Excel software. Statistical significance was assessed using Student's *t*-test to compare the vehicle control group with a drug-treated group. All the statistical tests were two-tailed. A *P* value less than 0.05 was considered to indicate a statistical significance.

## 3. Results

### 3.1. Effect of 0.5% DMSO on Angiogenesis in Zebrafish

**Figure 1:**
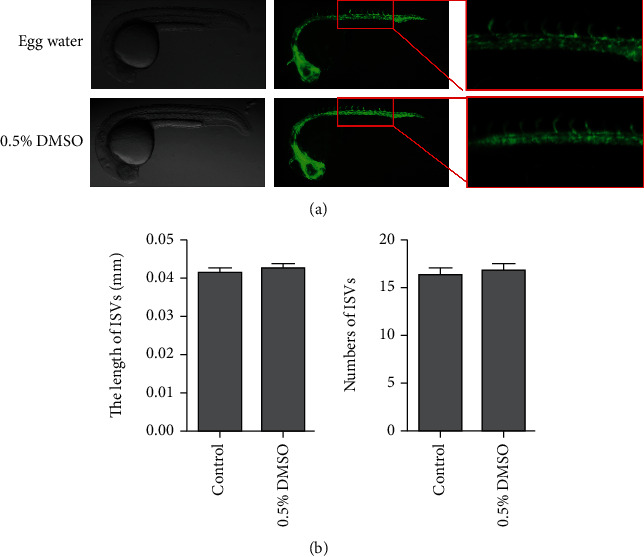
Effect of 0.5% DMSO on the growth of intersegmental vessels (ISVs) in the zebrafish model. (a) Bright-field and fluorescent images of zebrafish embryos at 24 h postfertilization (hpf) treated with egg water and 0.5% DMSO. (b) Average length and number of ISVs in zebrafish treated with different solvents.

To maximize drug dissolution, we used 0.5% DMSO as the drug carrier control. First, we test the effect of DMSO to angiogenesis in zebrafish. As shown in Figure 1, there was no significant difference in the length and number of ISVs in embryos treated with egg water and 0.5% DMSO.

### 3.2. Identification of Three Angiogenic Agents

**Figure 2:**
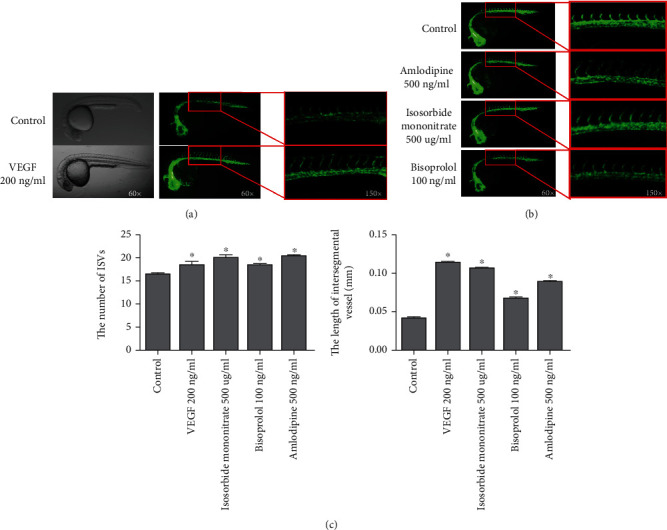
Chemical screen to identify angiogenic drugs in cardiovascular medicine. (a) Bright-field and fluorescent images of zebrafish embryos in 24 hpf treated with 0.5% DMSO (control) and 200 ng/ml VEGF (positive control) for 24 h. (b) Amlodipine-, isosorbide mononitrate-, and bisoprolol-treated embryos are shown. (c) Average ISV numbers and lengths in the control and drug-treated zebrafish embryos. Values for the average ISV numbers and lengths are present as mean ± SD. ^∗^*P* < 0.05 vs. control group.

To determine the proangiogenic properties of the drug, we used a transgenic Tg (fli1: EGFP) zebrafish as an animal model and 0.5% DMSO as the solvent of the drug. At the same time, VEGF, a known angiogenic compound, was used as a positive control. As a control, VEGF (200 ng/ml) was conducive to the formation of ISVs (Figure 2(a)). Then, as shown in Figure 2(b), 500 *μ*g/ml isosorbide mononitrate, 100 ng/ml amlodipine, and 500 ng/ml bisoprolol fumarate significantly promoted the growth of angiogenic ISVs in embryos (*P* < 0.05). The bar graph in Figure 2(c) showed the accurate length and number of ISVs after treatments with 3 drugs. These data strongly suggested that isosorbide mononitrate, amlodipine, and bisoprolol fumarate had angiogenic properties, depending on the concentrations.

### 3.3. Identification of Three Antiangiogenic Drugs

**Figure 3:**
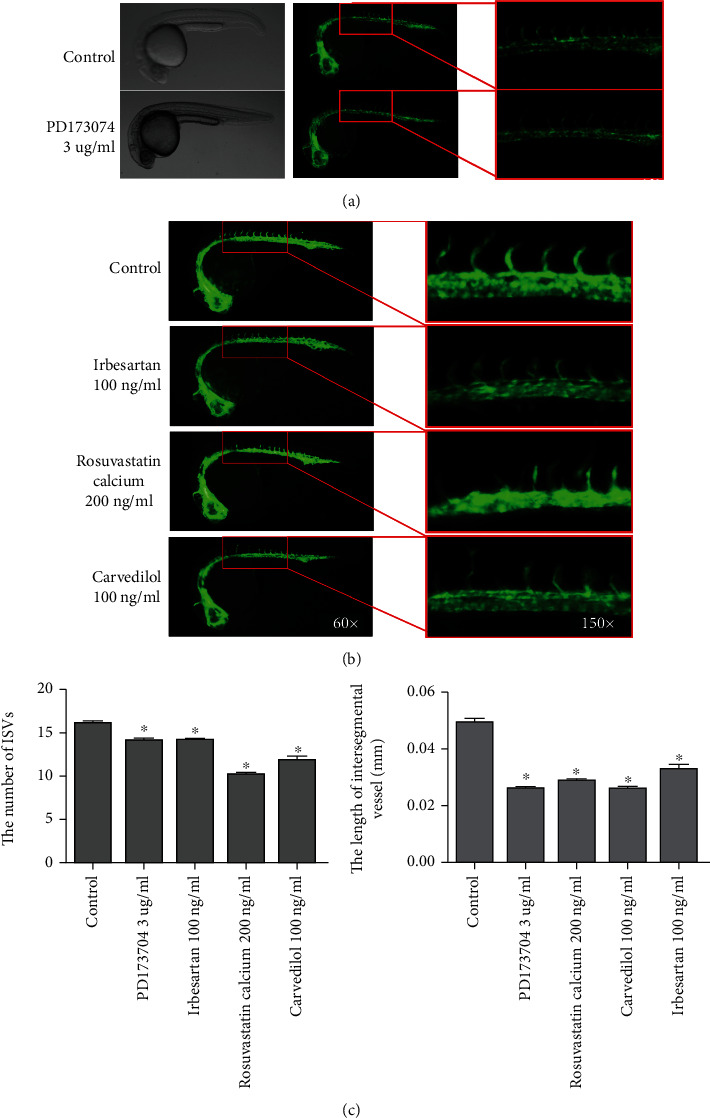
Effects of antiangiogenic drugs on ISV formation in Tg (flk1: EGFP) zebrafish embryos. (a) Bright-field and fluorescent images of zebrafish embryos at 24 hpf treated with 0.5% DMSO (control) and 3 *μ*g/ml PD173074 (positive control) for 24 h. (b) Fluorescent images of zebrafish embryos treated for 24 h with the antiangiogenic agents identified. Control: embryo treated with 0.5% DMSO. (c) Average number and length of ISVs was significantly inhibited, compared with the control, by the drugs at their optimal concentrations. Columns represent the mean of three independent experiments (*n* = 20). ^∗^*P* < 0.05 vs. control group.

In the process of drug screening, we also found that the other 3 drugs could inhibit the formation of ISVs. First, we used an antiangiogenic compound PD173074 as a positive control, which suppresses angiogenesis through inhibiting fibroblast growth factor receptor 1 (FGFR1) [12, 13]. PD173074 (3 *μ*g/ml) could significantly decrease the number and length of ISVs (Figure 3(a)).  Figure 3(b) showed the inhibitory effects of carvedilol, irbesartan, and rosuvastatin calcium on angiogenesis in Tg (flk1: EGFP) zebrafish embryo. In addition, the average length and number of ISV were significantly reduced after treatments with 100 ng/ml carvedilol, 100 ng/ml irbesartan, and 200 ng/ml rosuvastatin calcium compared to those in the control group (Figure 3(c), *P* < 0.05), suggesting that carvedilol, irbesartan, and rosuvastatin calcium could suppress the formation of capillary under similar experimental conditions.

### 3.4. Expression of VEGF in HUVEC Model *In Vitro*

**Figure 4:**
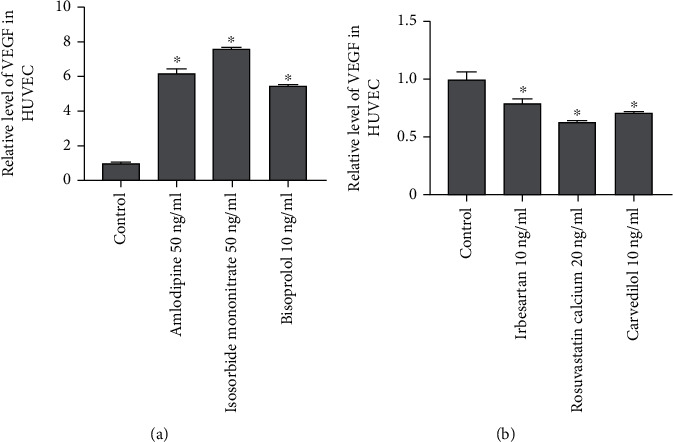
Expression of VEGF in HUVECs treated with different drugs which have an effect on the growth of ISV in zebrafish. VEGF expression level was detected by qRT-PCR. (a) Angiogenic agents significantly increased the expression level of VEGF in HUVECs. (b) The expression level of VEGF was significantly downregulated in HUVECs treated with an angiogenic agents. ^∗^*P* < 0.05 vs. control group.

Due to the potent pro- and antiangiogenic activities of drugs, its mechanism is worthy of further investigation. Among the known angiogenic factors, VEGF is the most important factor in the process of angiogenesis [14]. To further explore the involvement of VEGF in drug-induced angiogenesis, an *in vitro* HUVEC model was established using drug incubation for 24 hours. The expression of VEGF mRNA was detected by qRT-PCR. Results showed that the expression of VEGF was dramatically increased in HUVEC exposure to 50 *μ*g/ml isosorbide mononitrate, 10 ng/ml amlodipine, and 50 ng/ml bisoprolol fumarate (Figure 4(a), *P* < 0.05). However, compared with the control group, the expression of VEGF was decreased in HUVECs incubated with 10 ng/ml carvedilol, 10 ng/ml irbesartan, and 20 ng/ml rosuvastatin calcium (Figure 4(b), *P* < 0.05). These data indicated that the above drugs may play a role in promoting angiogenesis and antiangiogenesis through the VEGF signal pathway.

## 4. Discussion

Results of this study indicated that isosorbide mononitrate, amlodipine, and bisoprolol fumarate exerted angiogenic effects, whereas carvedilol, irbesartan, and rosuvastatin calcium inhibited angiogenesis. Besides, these drugs might regulate angiogenesis through the VEGF pathway.

Zebrafish has become a novel preclinical model that can support rapid decision-making in the early phases of the drug discovery process [15]. Our study demonstrated that zebrafish can high-throughput screen the effects of cardiovascular on vascular development by analyzing the phenotypic changes of zebrafish embryos after drug treatment. This model may be valuable for drug discovery when the pharmacological targets are unknown [16]. However, a stable zebrafish model for chemical screening is still under development. To fully validate the zebrafish model, more efforts need to be made [8].

In the transgenic zebrafish model, we found that isosorbide mononitrate, amlodipine, and bisoprolol fumarate might exert angiogenic effects through upregulating VEGF expression (Figures 2(b) and 4). As an organic nitrate vasodilator, isosorbide mononitrate can relax the peripheral vascular muscles by increasing nitric oxide (NO) release, thereby reducing systolic blood pressure [17]. Besides, NO could enhance angiogenesis through inducing VEGF expression by cyclic guanosine monophosphate pathway- (cGMP-) dependent pathway [18]. Furthermore, NO can promote cGMP production in HUVECs [19]. Thus, isosorbide mononitrate might stimulate angiogenesis through activating the NO-cGMP-VEGF pathway.

Amlodipine, a dihydropyridine calcium (Ca^2+^) channel blocker used for treatment of hypertension, also shows angiogenic activity in human coronary artery endothelial cells *in vitro* [20]. Moreover, amlodipine inhibitor suppresses angiogenesis in EA.hy926 endothelial cells [21]. However, the correlation between amlodipine and VEGF has not been reported. Therefore, this study revealed that amlodipine might induce angiogenesis by regulating VEGF for the first time.

Bisoprolol fumarate is a beta-selective blocker and an effective drug for the treatment of heart failure and hypertension. A previous study has demonstrated that bisoprolol fumarate displays angiogenic activity in mouse aortic ring assay [22]. Our results confirmed the effect of bisoprolol fumarate on angiogenesis in zebrafish model, suggesting that the zebrafish model could be used for the high-throughput screen of cardiovascular drugs. Besides, VEGF blockade prevents the effect of bisoprolol fumarate on angiogenesis [23], which is consistent with our results.

Furthermore, we also identified the antiangiogenic activity of carvedilol, irbesartan, and rosuvastatin calcium in the zebrafish model. Carvedilol is a pharmacological antioxidant with *α*1- and nonselective *β*-adrenoceptor antagonist activity, which is widely used in hypertension and heart failure [24]. However, previous studies only revealed the antiangiogenic activity of carvedilol in intrahepatic angiogenesis through the VEGF pathway [25, 26]. Thus, this study indicated the antiangiogenic activity of carvedilol in cardiovascular by zebrafish model.

Irbesartan is an angiotensin II receptor blocker. Recent research has shown that irbesartan may reduce angiogenesis by decreasing the number of infiltrating cells expressing VEGF in cancer [27]. Besides, irbesartan also inhibits coronary angiogenesis in rats [28]. Therefore, these studies further suggest that the zebrafish model could be used for the high-throughput screen of cardiovascular drugs.

Rosuvastatin calcium is a member of the statins family. Currently, the effect of statins on angiogenesis remains controversial. Weis et al. concluded that statins have a biphasic dose-dependent effect on angiogenesis [29]. In the zebrafish model, rosuvastatin calcium displayed the antiangiogenic effect at 10-200 ng/ml, which was similar to that reported by Wang et al. [12]. The discrepancy in currently available data can be attributed to differences in statins concentrations and applied animal models. In addition, rosuvastatin calcium regulates angiogenesis by modifying VEGF expression in rats [30, 31], which is consistent with our study. Thus, rosuvastatin calcium (10-200 ng/ml) might induce angiogenesis by regulating VEGF expression.

However, there were several limitations in this study. We did not address the problem of the potential effects of hydrophilicity that may affect drug absorption and lead to possible false negative results. In addition, like any other animal models, the zebrafish model does not reliably predict the human outcome; nevertheless, it provides valuable insights into the “new” pharmacological effects of the drugs tested. The evidence obtained from this model may enrich our understanding of the pharmacological profiles of the currently used cardiovascular drugs.

## 5. Conclusion

Results of this study indicated that isosorbide mononitrate, amlodipine, and bisoprolol fumarate exerted angiogenic effects, whereas carvedilol, irbesartan, and rosuvastatin calcium inhibited angiogenesis. Besides, these drugs might regulate angiogenesis through the VEGF pathway. The new functions of these drugs should improve the treatment of cardiovascular diseases.

## Figures and Tables

**Table 1:** Blood compound concentration in human.

Tested agents (CAS number)	Pharmacological class	Known pharmacological activities	Drug concentration range
Isosorbide mononitrate (16051-77-7)	Nitrate-class drug which can release nitric oxide and active cGMP in the endothelium cell.	Dilate the blood vessels to reduce blood pressure.	50-1000 *μ*g/ml
Digoxin (20830-75-5)	Decrease the function of the Na^+^/K^+^ATPase pump so as to raise the calcium concentration in myocardiocytes.	Slightly increase myocardial contractility and decrease the heart rate and blood pressure.	30-2000 ng/ml
Nitroglycerin (55-63-0)	Nitrate-class drugs.	Vasodilation widening of the blood vessels.	3-100 ng/ml
Bisoprolol fumarate (104344-23-2)	Selective type *β*_1_ adrenergic receptor blocker in the heart muscle cells and heart conduction tissue.	Less contractility of the heart muscle and lowered heart rate.	10-500 ng/ml
Metoprolol Tartrate (56392-17-7)	Selective *β*1 receptor blocker.	Decrease the heart rate and contraction.	0.05-100 *μ*g/ml
Amiodarone hydrochloride (19774-82-4)	Class III antiarrhythmic agent and prolongs phase 3 of the cardiac action potential.	Slows intracardiac conduction of the cardiac action potential.	0.5-4 *μ*g/ml
Lidocaine hydrochloride (137-58-6)	A common local anesthetic and antiarrhythmic drug.	Class 1B antiarrhythmic drug, decreases the ventricular rate.	1-100 *μ*g/ml
Propafenone hydrochloride (34183-22-7)	Slowing the influx of sodium ions into the cardiac muscle cells.	Decrease in excitability of the cardiac muscle cells.	500-2000 ng/ml
Amlodipine (88150-42-9)	Dihydropyridine class, a long-acting calcium channel blocker.	Relax the smooth muscle in the arterial wall, decreasing total peripheral resistance and hence reducing blood pressure.	5-1000 ng/ml
Carvedilol (72956-09-3)	Beta blockers (*β*1, *β*2) and alpha blocker (*α*1).	Slow the heart rhythm and reduce the force of the heart's pumping. Lower blood pressure and reduce heart failure.	10-200 ng/ml
Dopamine hydrochloride (62-31-7)	Catecholamine neurotransmitter.	Increased heart rate and blood pressure.	5-100 *μ*g/ml
Irbesartan (138402-11-6)	Angiotensin II receptor antagonists.	Modulate the renin–angiotensin-aldosterone system.	0.04-4 *μ*g/ml
Spironolactone (52-01-7)	Synthetic steroid.	Inhibits the effect of aldosterone and decreases the reabsorption of sodium and water.	0.1-1000 *μ*g/ml
Epinephrine hydrochloride (55-31-2)	A hormone and a neurotransmitter.	Increases heart rate and constricts blood vessels.	0.01-2 *μ*g/ml
Rosuvastatin Calcium (147098-20-2)	A member of the drug class of statins.	A competitive inhibitor of the enzyme HMG-CoA reductase, reduce the level of LDL cholesterol.	10-2000 ng/ml
Clopidogrel (113665-84-2)	Thienopyridine class antiplatelet agent, irreversibly inhibiting a receptor called P2Y12, an adenosine diphosphate ADP chemoreceptor.	Inhibit blood clots.	15-1000 *μ*g/ml
Ticlopidine (55142-85-3)	An antiplatelet drug in the thienopyridine family.	Inhibits platelet aggregation and prolongs bleeding time.	10-200 *μ*g/ml

## Data Availability

All data generated or analyzed during this study are included in this article. Further inquiries can be directed to the corresponding author.

## Abbreviations

ARB: Angiotensin II receptor blocker
Ca^2+^: Calcium
cGMP: Cyclic guanosine monophosphate pathway
CHD: Coronary heart diseases
CVD: Cardiovascular disease
GFP: Green fluorescence protein
DLAV: Dorsal longitudinal anastomotic vessel
DMSO: Dimethyl sulfoxide
HUVEC: Human umbilical vein endothelial cell
ISV: Intersegmental blood vessel
NO: Nitric oxide
qRT-PCR: Quantitative reverse transcriptase-polymerase chain reaction
VEGF: Vascular endothelial growth factor.
